# Two Cases of Respiratory Insufficiency Secondary to Pre-procedural Nerve Blocks for Upper Extremity Injuries

**DOI:** 10.7759/cureus.20511

**Published:** 2021-12-19

**Authors:** Nishil J Patel, Morghan Jameson, Matthew Leonard, Bracken Burns

**Affiliations:** 1 Osteopathic Medicine, Edward Via College of Osteopathic Medicine, Old Bridge, USA; 2 Medicine, Quillen College of Medicine, East Tennessee State University, Johnson City, USA; 3 Trauma, Ballad Health Trauma Services, Johnson City, USA; 4 Surgery, Quillen College of Medicine, East Tennessee State University, Johnson City, USA

**Keywords:** interscalene nerve blocks, interscalene, respiratory insufficiency, upper extremity trauma, respiratory failure

## Abstract

Interscalene nerve blocks are common procedures performed before upper extremity surgeries in order to provide post-op pain relief and improve recovery time. Here we present two cases of patients who underwent a unilateral supraclavicular and bilateral interscalene nerve block, respectively. The first patient had no risk factors but the second presented with a body mass index of 45.5 and a history of symptoms consistent with obstructive sleep apnea but never diagnosed. Both patients experienced some form of respiratory distress diagnosed via changes in chest x-ray and clinical presentation. The mechanism of injury that occurs in these procedures is typically from inadvertent damage to the phrenic nerve. Mild adverse effects in interscalene nerve block are relatively common. However, there is minimal data in regards to performing bilateral interscalene nerve blocks. The purpose of this study is to highlight that severe complication in both high and low-risk patients can occur but may be reduced with a safer approach and more effective communication among multidisciplinary team members.

## Introduction

Interscalene nerve blocks are commonly performed during surgical procedures that typically involve the shoulder, upper arm, or elbow. The procedure is most commonly performed by a Regional Anesthesia Specialist or an Anesthesiologist at smaller community hospitals [1]. The nerve blocks are usually accomplished by administering either bupivacaine or ropivacaine [1]. The mechanism of action involves blocking nerve impulses that lead to upper extremity numbness and weakness in the region innervated by the brachial plexus [2]. The benefits of interscalene nerve block include but are not limited to pain relief during the procedure for up to six hours, decreased time in the post-anesthesia care unit (PACU) [3], and postop pain relief for up to 24 hours in the absence of opioids [4]. However, there are some known side effects and potentially severe complications associated with interscalene nerve blocks. Pneumothorax, nerve damage, spinal cord trauma, and most commonly shortness of breath are side effects due to a misguided needle placement [5]. Diaphragmatic paralysis can occur due to transient phrenic nerve palsy from direct injury. Prolonged paresis is rare, with an incidence of 0.048% [3]. In addition, complications from an interscalene block are more common in patients with pulmonary comorbidities [1].

Certain studies indicate a decrease in adverse effects with guided ultrasound use, performing the procedure via a supraclavicular approach, using less local anesthetic volume, and improving interdisciplinary communication when dealing with high-risk patients. Therefore, it is important to consider safer approaches and improved communication methods to decrease the adverse-related events in high-risk patients indicated for an interscalene nerve block.

## Case presentation

The first patient is a 36-year-old male weighing 91 kg who presented with a closed fracture of the right ulna and radius as well as facial trauma to the left side. The patient was an unrestrained passenger that was ejected during a motor vehicle accident. The upper extremity injuries required open reduction and internal fixation of the right radius and ulna (ORIF). A portable chest x-ray upon arrival showed no evidence of acute cardiopulmonary process.

Prior to the operation, and after informed consent, an ultrasound-guided right-sided supraclavicular nerve block of 30 mL of 0.5% Bupivacaine was administered by the anesthesiologist. No immediate complications were recorded after the block or during surgery.

**Figure 1:**
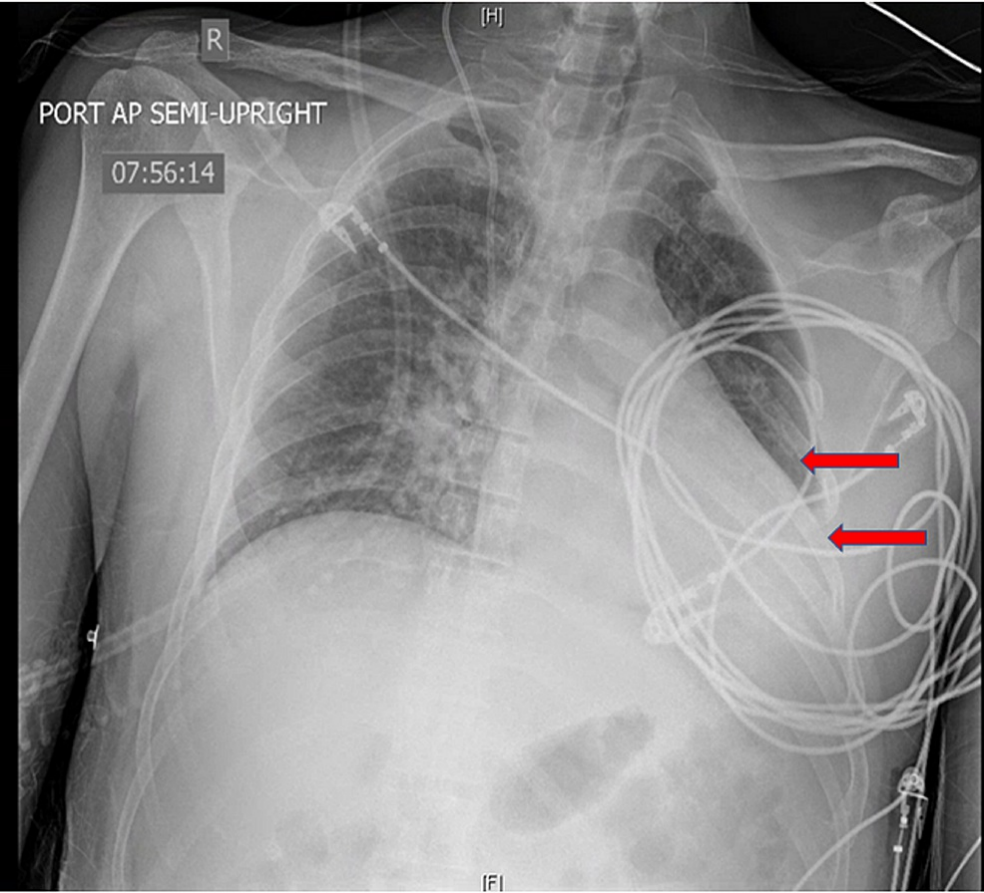
POD#1 portable chest x-ray showing low lung volumes with patchy areas of atelectasis. Arrows indicate low lung volume on left with the cardiac shift to left. POD - post-operative day

The patient was transferred to PACU where he subsequently developed respiratory distress. Due to respiratory rate as high as 48 breaths per minute, the patient was placed on bilevel positive airway pressure (BiPAP) and subsequently transferred to the surgical intensive care unit (SICU). After a few hours of monitoring in the SICU, the patient’s respiratory rate was in the 30s and the patient stated he could breathe much better. The patient was transitioned to nasal cannula from BiPAP on post-operative day 1 (POD#1). The respiratory rate at this time was 18. POD#1 portable chest x-ray showed low lung volume with persistent atelectasis and/or infiltrative changes in the left lung base (Figure 1).

**Figure 2:**
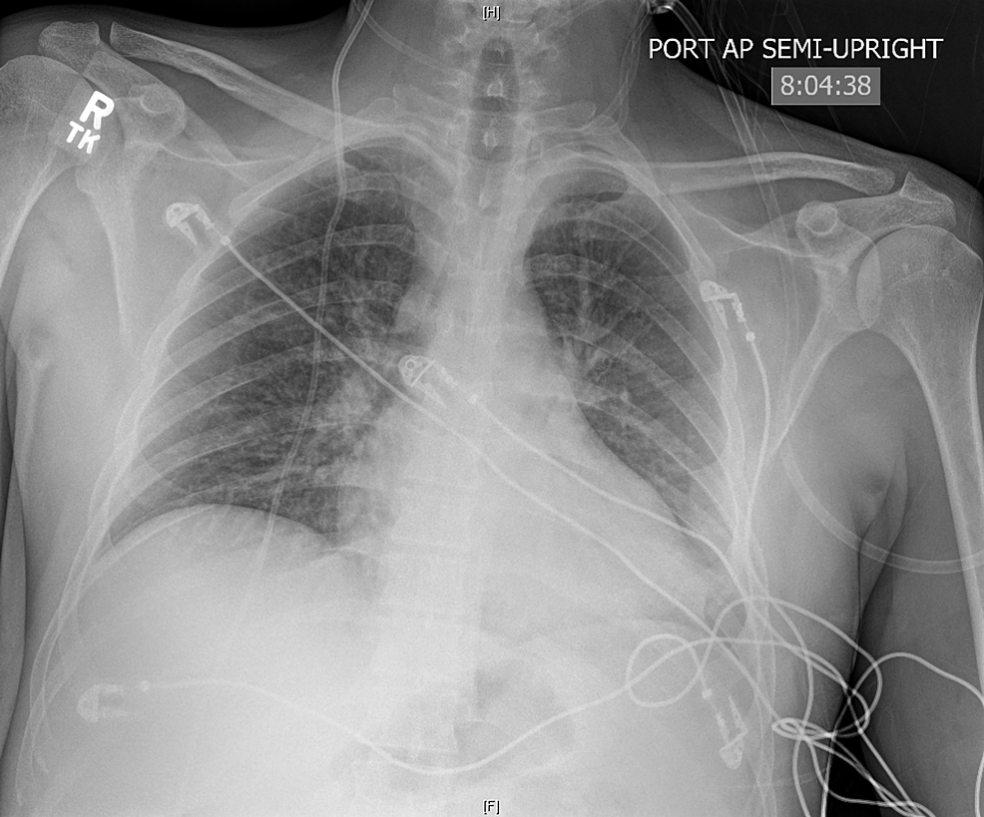
Portable chest x-ray day 4

A portable chest x-ray on day 4 showed no acute heart or lung disease appreciated (Figure 2). Incentive spirometry was encouraged, and the patient was able to be weaned off oxygen. On hospital day 5, the patient was discharged home with outpatient follow-up. 

**Figure 3:**
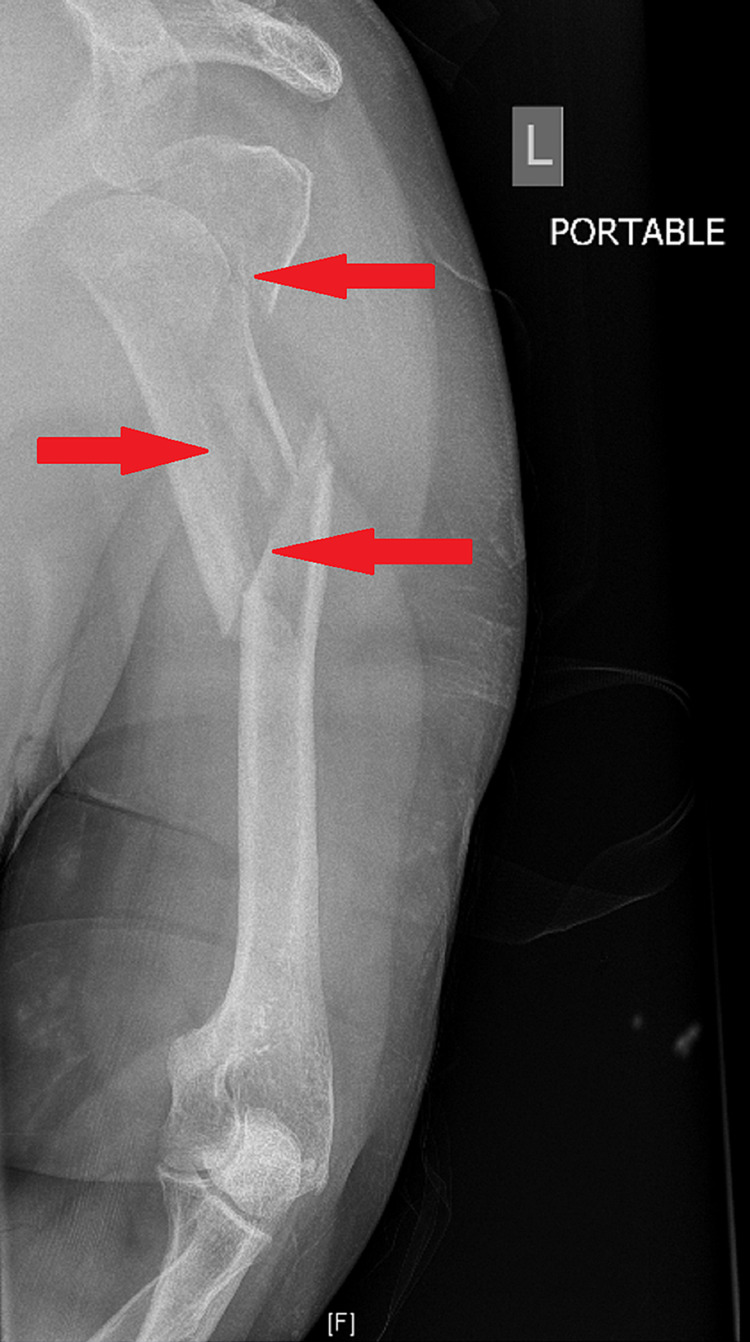
X-ray of LUE: Left humeral head, transverse fractures across the humeral neck with longitudinal fracture extending into the mid-shaft. Medial angulation of the proximal fracture fragments. Arrows indicate left humeral head, transverse fractures across the humeral neck with longitudinal fracture extending into the mid-shaft. Medial angulation of the proximal fracture fragments. LUE - left upper extremity

Patient two is a 56-year-old male, with a body mass index (BMI) of 45.5 and past medical history significant for symptoms consistent with obstructive sleep apnea who presented to the emergency department following a mechanical fall leading to bilateral humerus fractures. The patient initially presented with hypoxia which improved with the application of 4L of oxygen via nasal cannula. X-ray of the left upper extremity (LUE) showed a comminuted intra-articular fracture of the left humeral head, transverse fractures across the humeral neck with longitudinal fracture extending into the mid-shaft, and medial angulation of the proximal fracture fragments. The humeral head is laterally rotated and inferiorly displaced. The greater trochanter is separated from the humeral head and lies superior to the humeral head and the glenoid fossa. The scapula and clavicle are intact (Figure 3).

**Figure 4:**
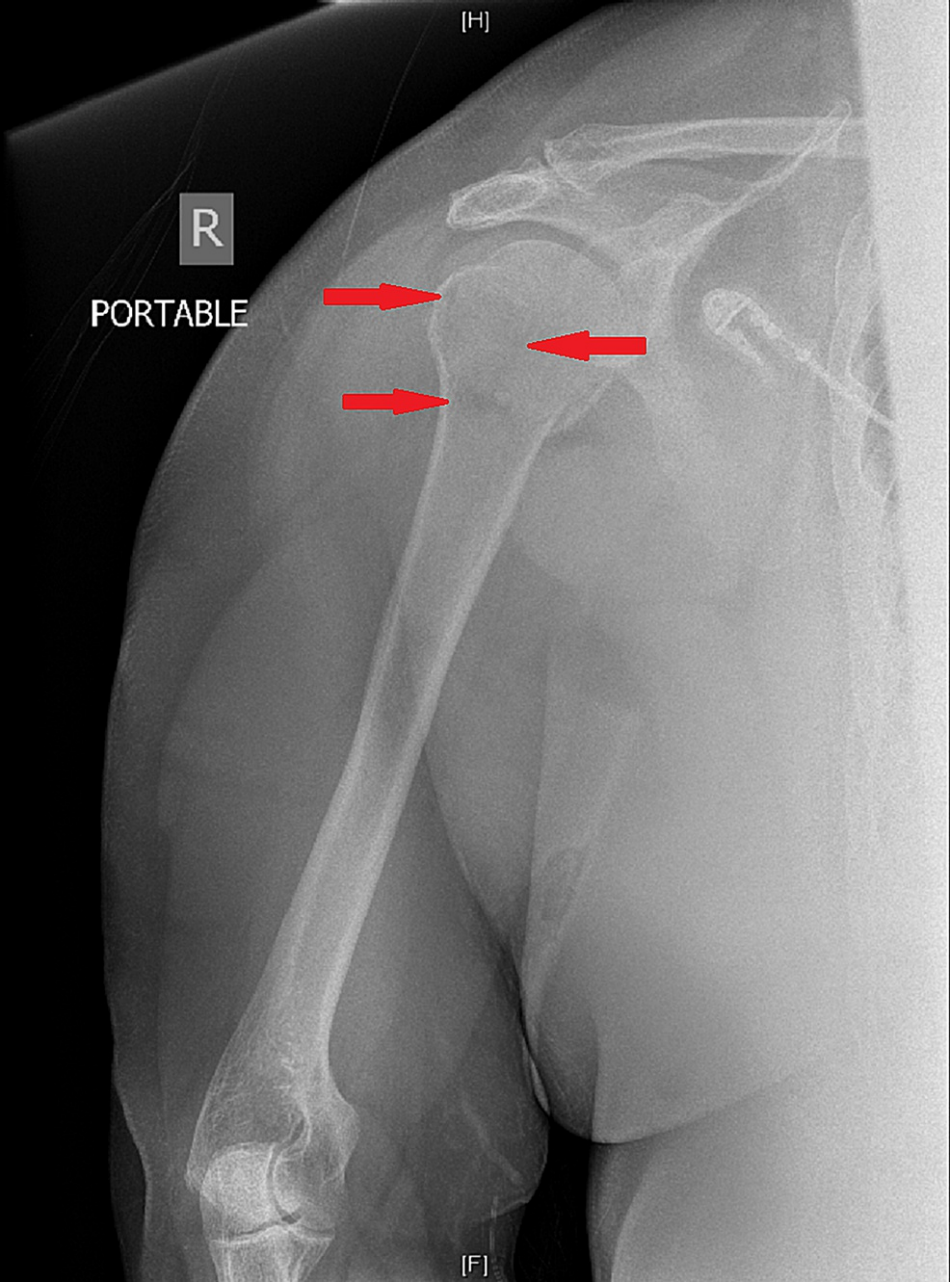
X-ray of RUE: Three-part proximal humeral fracture (arrows) RUE - right upper extremity

X-ray of the right upper extremity (RUE) showed a three-part proximal humeral fracture with components containing the greater tuberosity, the lesser tuberosity and at the surgical neck (Figure 4).

Prior to operation, a bilateral interscalene nerve block using 0.5% Ropivacaine 30mL was performed. The operating physician noted the patient was having trouble breathing when marking him in the pre-anesthesia area. Anesthesia was alerted, and the decision was made to intubate the patient due to respiratory distress secondary to bilateral blocks of the phrenic nerve. The patient was intubated uneventfully and ORIF of the bilateral humeri was completed with no acute events.

**Figure 5:**
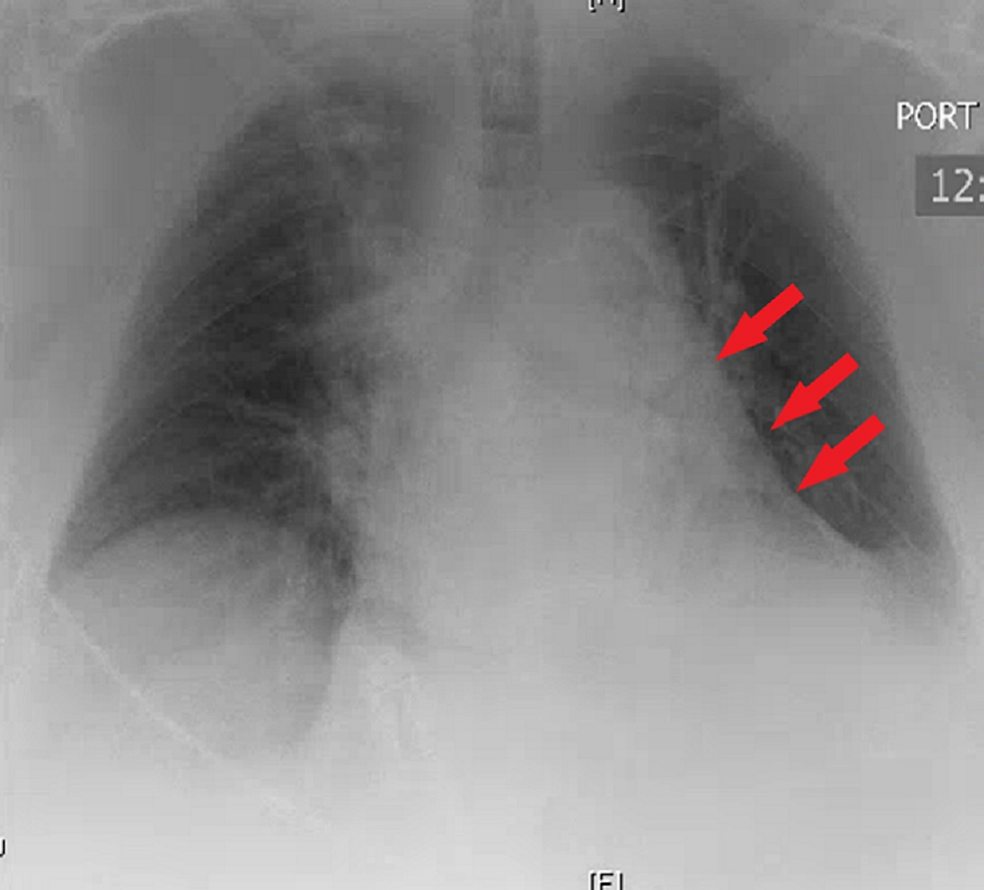
Post-operative x-ray demonstrating low left-sided lung volume (arrows).

After the operative intervention, the patient was extubated and taken to the PACU. In the PACU, the patient developed respiratory distress and was reintubated. Subsequently, the patient was transferred to the ICU for ongoing care. Chest x-ray at this time showed low lung volumes (Figure 5). Troponins were elevated to 0.46 on the day of surgery but trended back down to 0.20 POD#1. On POD#1, the patient's respiratory mechanics were evaluated and the patient was successfully extubated. On the following day, the patient was transferred to the floor. Based on physical therapy evaluation, the patient was discharged to rehab on hospital day 5.

## Discussion

Case number one highlights that respiratory failure is a possible outcome following a supraclavicular nerve block. The second case was a high-risk patient that developed respiratory failure from bilateral interscalene nerve blocks. The second patient was considered high risk given his high BMI and history of obstructive sleep apnea symptoms. Both cases had respiratory post-op complications but different mechanisms in which the respiratory insufficiency/failure occurred. After a multidisciplinary review of both cases, it was felt that scalene nerve block was the major contributing factor in these patients' respiratory failure. Typically, nerve blocks for upper extremity procedures are performed using the interscalene approach, but the supraclavicular approach may be used to produce complete anesthesia of the upper extremity and has reported fewer adverse effects [4]. Even though the supraclavicular approach can produce a more robust motor blockade, it could still inadvertently lead to hemidiaphragmatic paresis [5]. The first case highlights a presentation of acute hypoxia which resulted from the supraclavicular approach. The transition of care from the operating room (OR) to the PACU and subsequently to the ICU are critical transitions in the patient’s care, and effective communication is one of the biggest challenges. Prior to the operation, this patient was on a medical-surgical floor and ultimately required SICU admission. During the transition from the PACU to the SICU, no direct communication occurred between the Anesthesia team and the SICU team. There was a study that reported anesthesiologists failing to transfer all of the important information in 67% of OR to PACU handoffs [6]. Lack of complete information being handed off to ICU nurses and physicians can harm the patient. In order to avoid major patient complications, there is a need for detailed and accurate communication of patient information during patient transfer among multidisciplinary teams [7]. Some best practices that have been shown to improve the transfer of care include but are not limited to appropriate personnel at the bedside for handoff, standardized checklists and scripts, and double verification of post-op orders [7]. The benefits of effective communication and appropriate sign-out between areas of patient care can help avoid negative outcomes. As a result of the performance improvement review of these two cases, our SICU and Anesthesia teams are developing a standardized bedside hand-off procedure. 

In the second case, a bilateral interscalene block was performed on a high-risk patient. Normally, bilateral interscalene nerve block is avoided in high-risk patients, but it was indicated in this patient due to bilateral humeri fractures. There were numerous concerning factors for this patient indicating an increased risk for potential adverse effects. Patients with predisposed respiratory insufficiency have a higher likelihood of developing ipsilateral phrenic nerve block leading to an approximately 25% decrease in pulmonary function [1]. Knowing this, closer respiratory monitoring and precautionary measures should be taken in patients that present with symptoms or diagnosis of obstructive sleep apnea. In addition, the possible need for emergent ventilatory support should always be considered when a bilateral block is being performed. If a bilateral block is performed, it is important to convey this information during the transition in areas of care. Some other recommendations to reduce potential complications are reducing the volume of local anesthetic injected, using the supraclavicular approach, or performing a shoulder block [3,8]. In one study using different anesthetic volumes, there was a noticeable difference in the incidence of paradoxical diaphragmatic movement; 45% in the low-volume group compared to 100% in the standard-volume group. The low volume and standard-volume group used 5 and 20mL, respectively [9]. Another study demonstrated the efficacy of shoulder block as compared to interscalene block with fewer complications; there was a significantly decreased incidence of dyspnea and muscle weakness [8].

## Conclusions

These two cases presented different types of pre-procedural nerve blocks that resulted in similar respiratory complications. Although the complications that resulted in both patients did not have long-term consequences we identified opportunities for better communication during transitions of care in both cases. Care should be taken to assess patient risk prior to scalene nerve blocks and when utilized standardized communication between transitions in care is recommended.

## References

[1] Zisquit J, Nedeff N (2021). Interscalene Block. https://pubmed.ncbi.nlm.nih.gov/30137775/.

[2] Vaid VN, Shukla A (2018). Inter scalene block: revisiting old technique. Anesth Essays Res.

[3] Koogler AT, Kushelev M (2018). Ultrasound-guided interscalene catheter complicated by persistent phrenic nerve palsy. Case Rep Anesthesiol.

[4] Chaudhuri S, Gopalkrishna M, Paul C, Kundu R (2012). Can bilateral bronchospasm be a sign of unilateral phrenic nerve palsy after supraclavicular brachial plexus block?. J Anaesthesiol Clin Pharmacol.

[5] Patel PR, Bechmann S (2020). Elevated Hemidiaphragm.. https://www.ncbi.nlm.nih.gov/books/NBK559255/.

[6] McElroy LM, Collins KM, Koller FL, Khorzad R, Abecassis MM, Holl JL, Ladner DP (2015). Operating room to intensive care unit handoffs and the risks of patient harm. Surgery.

[7] Wheeler DS, Sheets AM, Ryckman FC (2018). Improving transitions of care between the operating room and intensive care unit. Transl Pediatr.

[8] Pani N, Routray SS, Pani S, Mallik S, Pattnaik S, Pradhan A (2019). Post-operative analgesia for shoulder arthroscopic surgeries: a comparison between inter-scalene block and shoulder block. Indian J Anaesth.

[9] Riazi S, Carmichael N, Awad I, Holtby RM, McCartney CJ (2008). Effect of local anaesthetic volume (20 vs 5 ml) on the efficacy and respiratory consequences of ultrasound-guided interscalene brachial plexus block. Br J Anaesth.

